# Risk factors for active tuberculosis in 938 QuantiFERON-positive schoolchildren in Mongolia: a community-based cross-sectional study

**DOI:** 10.1186/s12879-019-4160-7

**Published:** 2019-06-17

**Authors:** Davaasambuu Ganmaa, Polyna Khudyakov, Uyanga Buyanjargal, Delgerekh Baigal, Munkhzul Baatar, Nomin Enkhamgalan, Sumiya Erdenebaatar, Batbayar Ochirbat, Buyankhishig Burneebaatar, Enkhtamir Purevdorj, Yanjindulam Purevsuren, Gantsetseg Garmaa, Erdenetuya Ganbaatar, Adrian R. Martineau

**Affiliations:** 1 Harvard T.H. Chan School of Public Health, Building 2, Room 211, 655 Huntington Ave, Boston, MA 02115 USA; 2 Mongolian Health Initiative, Royal Plaza, Bayanzurkh District, Ulaanbaatar, Mongolia; 3 Mongolian National Health Sciences University, Ulaanbaatar, Mongolia; 4 National Center for Communicable Disease, Ulaanbaatar, Mongolia; 5 Blizard Institute, Barts and The London School of Medicine and Dentistry, Queen Mary University of London, E1 2AB, London, UK

**Keywords:** Tuberculosis, Children, Risk factors, *QuantiFERON®-TB gold*, *Vitamin D*

## Abstract

**Background:**

There is controversy regarding the relative influence of ‘exogenous’ versus ‘endogenous’ factors on the risk of progression from latent tuberculosis infection to active tuberculosis (TB) disease in children.

**Methods:**

We conducted a cross-sectional analysis to identify risk factors for active tuberculosis in *QuantiFERON®-TB Gold (QFT-G)-positive* children aged 6–13 years attending 18 schools in Ulaanbaatar, Mongolia. Children underwent clinical and radiological screening for active tuberculosis, and data relating to potential risk factors for disease progression were collected by questionnaire and determination of serum 25-hydroxyvitamin D (25[OH]D) concentrations. Risk ratios were calculated using generalized estimating equations with adjustment for potential confounders.

**Results:**

129/938 (13.8%) QFT-positive children were diagnosed with active tuberculosis. Risk of active tuberculosis was independently associated with household exposure to pulmonary TB (adjusted risk ratio [aRR] 2.40, 95% CI 1.74 to 3.30, *P* < 0.001), month of sampling (adjusted risk ratio [aRR] for March–May vs. June–November 3.31, 95% CI 1.63 to 6.74, P < 0.001; aRR for December–February vs. June–November 2.53, 95% CI 1.23 to 5.19, *P* = 0.01) and active smoking by the child (aRR 5.23, 95% CI 2.70 to 10.12, P < 0.001). No statistically significant independent association was seen for age, sex, socio-economic factors, presence of a Bacillus Calmette–Guérin (BCG) scar, tobacco exposure or vitamin D status.

**Conclusions:**

Household exposure to active TB, winter or spring season and active smoking were independently associated with risk of active tuberculosis in QFT-positive children. Our findings highlight the potentially high yield of screening child household contacts of infectious index cases for active tuberculosis in low- and middle-income countries.

## Background

Around one million children develop active tuberculosis each year, and more than 200,000 die of the disease, ranking it in the top ten causes of child mortality worldwide [1, 2]. Effective control of tuberculosis in children is therefore a public health priority [3]. Cross-sectional studies evaluating determinants of disease risk can inform the design of tuberculosis control programmes by identifying risk factors for disease that are potentially amenable to intervention.

Development of active tuberculosis in childhood can be regarded as a two-stage process, in which acquisition of asymptomatic infection with *Mycobacterium tuberculosis* is followed by progression to symptomatic active disease. The risk factors governing each stage may be distinct, with the former step regarded by some as being driven by ‘exogenous’ risk factors (such as intensity of exposure to an infectious index case), with the latter step being primarily influenced by ‘endogenous’ risk factors (such as age) [4, 5]. Investigation of factors influencing disease progression in children tend to focus on household contacts, as the yield of cases is relatively high: although this approach affords greater statistical power for a given sample size, it precludes the possibility of investigating whether household exposure to an index case is a risk factor for disease progression as well as for acquisition of infection, as is well-recognized [6]. By comparison, there are relatively few community-based studies investigating risk factors for disease progression in childhood. Where such studies have been conducted, the diagnosis of *M. tuberculosis* infection is often based on tuberculin skin testing (as opposed to an Interferon-γ release assay, or IGRA) which can yield false-positive results in individuals who are sensitized to BCG or environmental mycobacteria [7].

We studied risk factors related to progression of *Mycobacterium tuberculosis* (MTB) infection among school aged children in Ulaanbaatar (UB), the capitol of Mongolia. As part of a clinical trial of vitamin D supplementation for prevention of TB infection [8], we screened 9814 children using the QuantiFERON Gold test (QFT-G), of whom 938 tested positive, and underwent clinical and radiographic screening for active TB which was diagnosed in 129 cases. For all QFT-G-positive individuals we collected comprehensive data relating to potential risk factors for disease progression, and we performed multivariable analyses to identify those that were independently associated with increased risk of active tuberculosis.

## Methods

### Study design and setting

Mongolia is a land -locked country with Russia to its north, and China to its east, south and west. Mongolia itself is thinly populated with only 3.1 million people. The capital, Ulaanbaatar, however, is densely populated, containing 46% (1.3 million) of the population. Secondary education is compulsory for children between the ages of 6 and 16.

TB is common in Mongolia. The World Health Organization (WHO) has estimated the incidence of active TB to be 428 per 100,000 people per year [9]. The prevalence of HIV infection is low, estimated at 0.02% of the population [10].

The study reported herein was approved by the Ethical Review Boards of the Mongolian Ministry of Health and the Mongolian National University, and by the Office of Human Research Administration at the Harvard T.H. Chan School of Public Health in Boston, MA, USA (IRB ref. no. 14–0513).

### Participants

These children were enrolled as part of the clinical trial of vitamin D supplementation in TB prevention with broad eligibility criteria. Children between the ages of 6 and 13, whose parents gave written consent, and who themselves gave written assent, were enrolled, unless: 1) the family planned to move away from UB within the next four years; 2) they were taking over 10 micrograms per day of any form of vitamin D; 3) they showed evidence of rickets on physical exam (leg bowing, knock knees, pectus carinatum, thickened wrists or ankles; 4) they were known to be seropositive for Human Immunodeficiency Virus (HIV) or to have hyperparathyroidism, sarcoidosis, or previous latent or active TB;, 5) they were taking cytotoxic or immunosuppressant medication, enzyme-inducing anticonvulsants or cardiac glycosides.

### Procedure

We collected the following information from parents or legal guardians, using an electronic questionnaire connected to the REDCap database: age, gender, monthly household income, type and ownership of residence, highest education level attained by either parent, number of people sleeping per room, smoking by household members and the children themselves, and exposure to a case of pulmonary TB present in the household at any time during the child’s lifetime.

Height and weight were measured with light clothing, but no shoes, hair ornaments or hats. A portable stadiometer (SECA, Hamburg, Germany) was used to measure height to the nearest 0.1 cm. A digital floor scale (SECA) was used to measure weight to the nearest 0.1 kg. Body Mass Index (BMI) was calculated as weight (kg) divided by the square of the height (m). Parameters of body composition; impedance, % body fat and fat-free mass, were measured using a body composition analyzer (SC-331S, Tanita, Tokyo, Japan). Children were examined for evidence of a BCG scar.

We used QuantiFERON-TB Gold High Altitude tubes (Qiagen, Hilden, Germany) to collect 3 ml of venous blood, which we processed according to the manufacturer’s instructions by the Global Laboratory (G-Lab) in UB, which participates in the QuantiFERON Quality Assurance Program of the Royal College of Pathologists in Australia. Children with positive test results were sent to physicians at the Mongolian National Centre for Communicable Diseases (NCCD) for clinical and radiological assessment for active TB, which was diagnosed using published criteria [11]. Facilities for mycobacterial culture were not available. Those diagnosed with active TB were treated at NCCD clinics, or District TB Dispensaries with 2 months of isoniazid, rifampicin, pyrazinamide and ethambutol, followed by 4 months of isoniazid and rifampicin. Response to treatment was based on a clinical and radiological evaluation over the course of anti-TB treatment. Specifically, a ‘good response’ was evidenced by a) reduced cough, b) resolution of pyrexia, c) increased weight, d) increased playfulness /activity reported by parents or caregivers, and/or e) improved chest radiograph appearance (in cases where this was abnormal at baseline).QuantiFERON-positive children in whom active TB was excluded were not preventively treated for latent tuberculosis infection, in line with Mongolian National TB Program (NTP) (Minister order #306) and WHO recommendations [12].

25-hydroxyvitamin D levels were measured using an enzyme linked fluorescent assay (VIDAS 250H Vitamin D total; Biomerieux, Marcy-l’Etoile, France). The assay was accredited by the Vitamin D External Quality Assessment Scheme (DEQAS). The total Coefficient of Variation (CV) was 7.9%, mean bias was 7.7% and the limit of quantitation (LOQ) was 8.1 ng/ml. We standardized non-zero 25(OH) D values using a set of 40 DEQAS serum samples as previously described [13]. Values below the LOQ were imputed as 5.7 ng/ml (i.e. the limit of quantitation divided by the square root of two). We then calculated deseasonalized (season-adjusted) values for each participant from their individual standardized 25(OH) D concentration and date of blood sample collection as described elsewhere [14].

### Statistical analysis

SAS software (version 9.4; SAS Institute, Cary, NC USA), STATA (version 15; StataCorp, College Station, TX USA) and Prism (version 7.03; GraphPad Software, San Diego, CA USA) were used to analyze data. We used a Wilson procedure without correction for continuity [15] to calculate 95% confidence intervals (CI) for the estimate of disease prevalence in QFT-G-positive children. Two multivariable analyses were conducted to identify factors that were independently associated with risk of active TB. Models were first adjusted for age and sex only. In the other models we additionally adjusted for all covariates that were associated with risk of active tuberculosis with *P* < 0.20 in the age- and sex-adjusted analysis, namely type of residence (centrally heated; not centrally heated; yurt), parental education (university/polytechnic; secondary school or lower), monthly household income, number of people per room, month of sampling, presence vs. absence of indoor smokers in the household, active smoking by the child, body mass index, % body fat, household exposure to an index case of pulmonary tuberculosis (PTB) and vitamin D deficiency, defined as serum 25(OH) D concentration < 10 ng/ml. We pre-specified this threshold based on findings of a meta-analysis reporting susceptibility to tuberculosis to be increased below this cut-off [16]. Risk ratios for the association between these independent variables and risk of active tuberculosis were estimated using generalized estimating equations (GEE) with the binary distribution, log link function and exchangeable working correlation structure [17]. We used Log-Poisson models when the log-binomial model failed to converge, which provide consistent but not fully efficient estimates of the risk ratio and its confidence intervals, [18]. We calculated Population Attributable Fraction (PAFs) and their 95% confidence intervals for potentially modifiable independent risk factors for active TB using STATA as previously described [19].

## Results

**Fig. 1:**
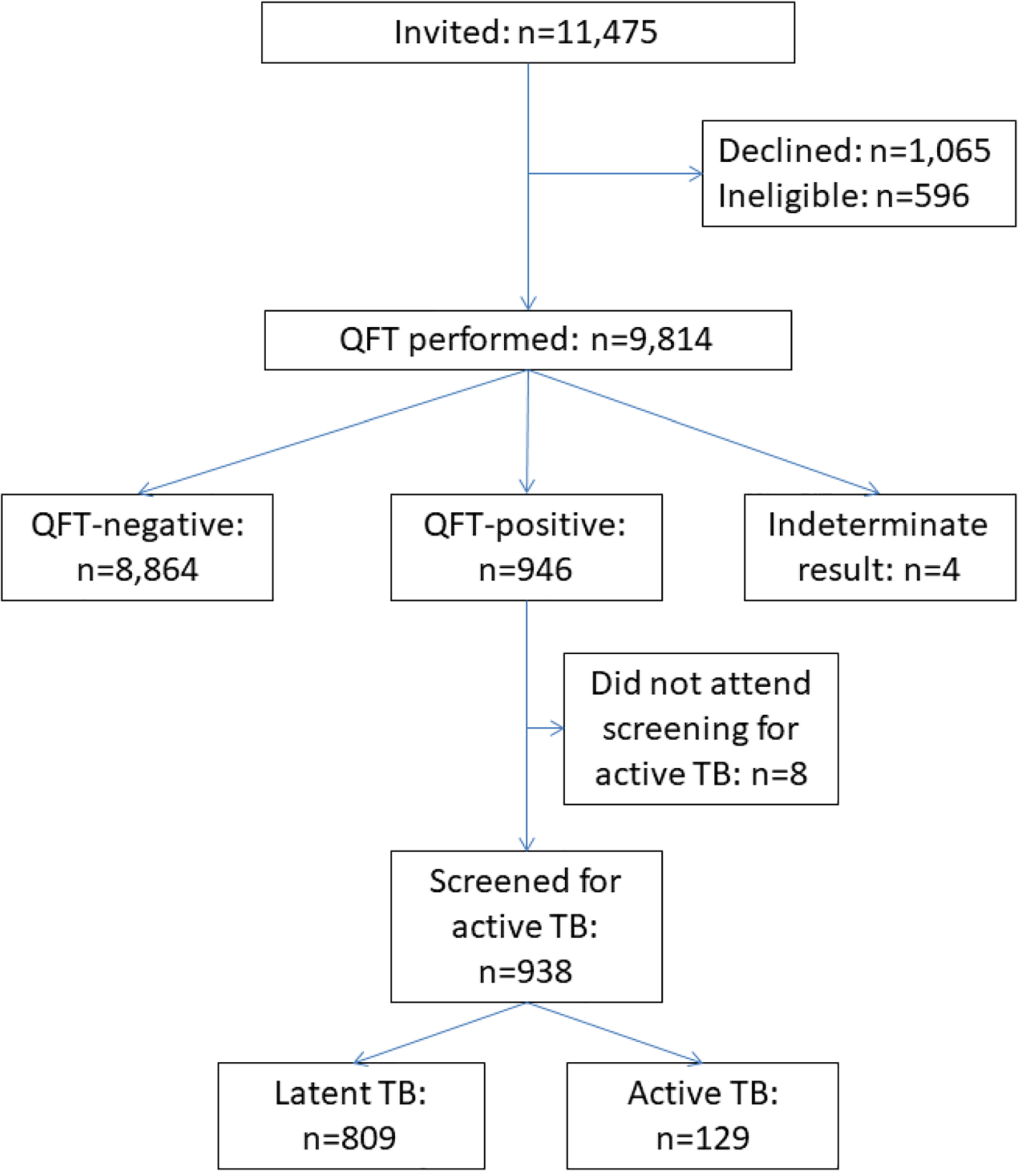
Participant flow

Between July 2015 to January 2017 a total of 11,475 children were invited to participate in the study, of whom 1065 (9.3%) declined and 596 (5.2%) were ineligible (Fig. 1). We performed the QFT-G test for the remaining 9814 children. Of those, 946 had positive QFT-G test results, 938 of whom were screened for active TB (Fig. 1).

Characteristics of the study population are presented in Table 1. Their mean age was 9.8 years, and 490 (52.2%) were female. Only 17.8% (167) lived in a house or apartment with central heating, 39.6% (373) lived in a house or apartment without central heating, and 42.4% (398) lived in a traditional Mongolian circular structure (usually felt covered) known as a ger (yurt in Russian usage). The mean family income was equivalent to US$ 779 per month. Household exposure to a PTB case was reported by 16.8% (158). At least one family member smoked tobacco indoors for 45.3% (408) of the participants. A total of 79.4% (745) had BCG scars. The BCG strains in use in Mongolia during the birth years of these children were: Japan BCG [2001–2003], Intervax Toronto [2003–2006], and SI India [2007–2009]).

**Table 1:** Participant characteristics (*n* = 938)

Characteristic		Number (%)/Mean (s.d.)
Mean age, years (s.d.)		9.8 (1.6)
Sex	Female, *n* (%)	490 (52.2)
	Male, *n* (%)	448 (47.8)
Parental education^(a)^	University/polytechnic, *n* (%)	127 (13.5)
	Secondary school or lower, *n* (%)	811 (86.5)
Type of residence	Centrally heated, *n* (%)	167 (17.8)
	Not centrally heated, *n* (%)	373 (39.8)
	Ger (Yurt), *n* (%)	398 (42.4)
Mean monthly household income, US dollars (s.d.)^(b)^		779 (592)
Home ownership	No, *n* (%)	220 (23.5)
	Yes, *n* (%)	718 (76.5)
Mean number of people/room (s.d.)^(b)^		3.5 (1.8)
No. of smokers in household^(c)^	0	530 (56.5)
	1 or more	408 (43.5)
Child actively smoking	No, *n* (%)	936 (99.8)
	Yes, *n* (%)	2 (0.2)
BCG scar	Absent	193 (20.6)
	Present	745 (79.4)
Mean body mass index, kg/m^2^ (s.d.)		17.1 (2.8)
Mean % body fat^(c)^ (s.d.)		17.7 (6.1)
Household PTB contact	No, *n* (%)	780 (83.2)
	Yes, *n* (%)	158 (16.8)
De-seasonalized serum 25(OH)D^(b)^	< 10 ng/ml, *n* (%)	281 (30.1)
	≥10 ng/ml, *n* (%)	652 (69.9)

Abbreviations: 25(OH) D, 25-hydroxyvitamin D; PTB, pulmonary tuberculosis; s.d., standard deviation; US, United States. ^a^highest educational level attained by either parent; ^b^missing values (income, 8 missing; number of people per room, 1 missing; 25OHD, 5 missing); ^c^Defined as a household member other than the participating child smoking tobacco indoors

Seasonally adjusted 25(OH) D levels were available for 933 of the 938 (99.5%), of whom 30.1% (281) had levels in the deficient range of less than 10 ng/ml.

Of the 938 QFT-positive children who underwent clinical and radiological screening, active TB was diagnosed in 129 and excluded in 809; prevalence of active TB among QFT-positive children was 13.8% (95% CI 11.7 to 16.1%). Details of symptoms, signs and chest radiographic appearances of children diagnosed with active TB are presented in Table 2: the majority (80.6%) were asymptomatic with no abnormality on clinical examination. All had abnormal chest radiograph appearances: hilar/mediastinal lymphadenopathy was observed in 125/129 (96.9%), parenchymal/bronchial involvement was present in 109/129 (84.5%), and pleural effusion was seen in 3/129 (2.3%). One hundred and nine children completed treatment and responded to it; 20 children were lost to follow-up before the end of TB treatment.

**Table 2:** Clinical and radiological features of children diagnosed with active TB (*n* = 129)

Characteristic		Number (%)
Principal symptom/sign of active TB	None	104 (80.6)
	Cervical lymphadenopathy	10 (7.8)
	Systemic symptoms only	5 (3.9)
	Cough	7 (5.4)
	Chest pain	3 (2.3)
Chest radiograph appearance	Hilar/mediastinal lymphadenopathy with parenchymal/bronchial involvement	107 (82.9)
	Hilar/mediastinal lymphadenopathy without parenchymal/bronchial involvement	17 (13.2)
	Hilar/mediastinal lymphadenopathy + pleural effusion	1 (0.8)
	Pleural effusion only	2 (1.6)
	Parenchymal involvement only	2 (1.6)
Response to anti-TB therapy	Good response	109 (84.5)
	Not known (lost to follow-up)	20 (15.5)

**Fig. 2:**
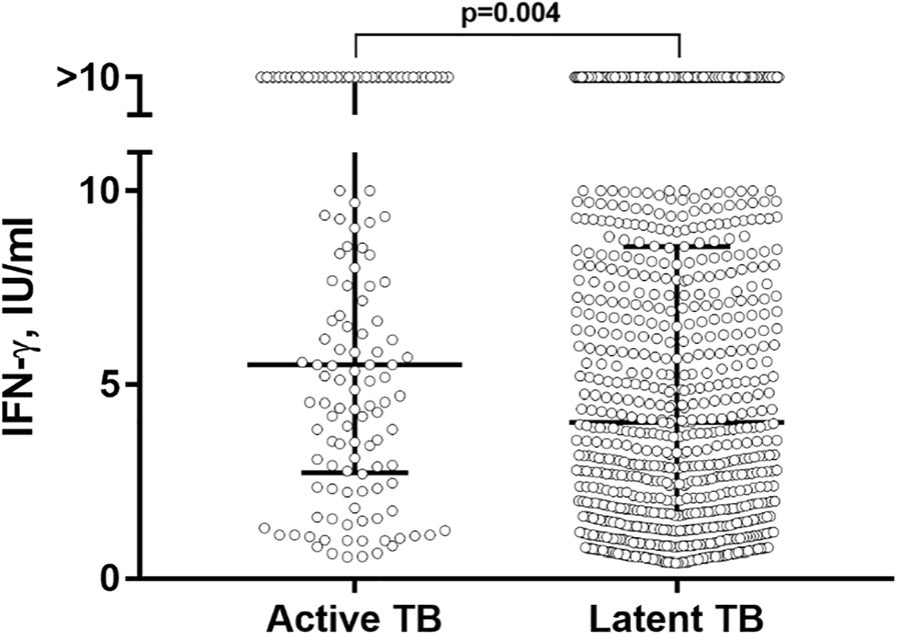
Antigen-stimulated IFN-γ, active vs. latent tuberculosis. *P* value from Mann Whitney test. Bars and whiskers show medians and interquartile ranges, respectively

Table 3 presents results of univariable and multivariable analyses evaluating potential determinants of active tuberculosis in the 938 QFT-positive participants who underwent screening for active TB. The fully adjusted model included age, sex, parental education, type of residence, monthly household income, number of people per room, month of sampling, presence vs absence of indoor smokers in the household, child’s active smoking status, body mass index, % body fat, household PTB contact and deseasonalized serum 25(OH) D concentration as covariates. The following risk factors were found to associate with risk of active TB after adjustment for these potential confounders: household exposure to pulmonary TB (adjusted risk ratio [aRR] 2.40, 95% CI 1.74 to 3.30, *P* < 0.001), month of sampling (aRR for March–May vs. June–November 3.31, 95% CI 1.63 to 6.74; aRR for December–February vs. June–November 2.53, 95% CI 1.23 to 5.19) and active smoking by the child (aRR 5.23, 95% CI 2.70 to 10.12). Population attributable risk fractions for potentially modifiable risk factors were 20.9% (95% CI 11.8 to 29.0%) for household exposure to pulmonary TB and 0.6% (− 0.4 to 1.6%) for active smoking by the child. No independent associations with risk of active tuberculosis were seen for sex, age, parental education, type of residence, household income, home ownership, number of people per room, number of tobacco smokers in the household, presence of a BCG scar, body mass index, % body fat or vitamin D status. The median IFN-γ concentration in supernatants of antigen-stimulated whole blood was slightly higher among children with active vs latent TB (5.51 vs 4.03 IU/ml, *P* = 0.004; Fig. 2).

**Table 3:** Risk factors for active tuberculosis in QuantiFERON®-TB Gold-positive schoolchildren (*n* = 938)

		Proportion with active tuberculosis	Univariable analysis		Adjusted for age and sex only		Adjusted for age, sex and covariates with *P* < 0.2 on age- and sex-adjusted analysis^(a)^	
			Relative risk (95% CI)	*P*	Adjusted relative risk (95% CI)	*P*	Adjusted relative risk (95% CI)	*P*
Sex	Female	64/490 (13.1%)	1.00 (ref)	–	1.00 (ref)	–	1.00 (ref)	–
	Male	65/448 (14.5%)	1.11 (0.81, 1.53)	0.52	1.13 (0.82, 1.55)	0.46	0.94 (0.66, 1.34)	0.74
Age			0.93 (0.84, 1.03)	0.14	0.92 (0.83, 1.02)	0.13	0.91 (0.81, 1.05)	0.13
Parental education^(b)^	University/polytechnic	10/127 (7.9%)	1.00 (ref)	–	1.00 (ref)	–	1.00 (ref)	–
	Secondary school or lower	119/811 (14.7%)	1.86 (1.00, 3.46)	0.048	1.94 (1.04, 3.60)	0.04	1.03 (0.56, 1.89)	0.91
Type of residence	Centrally heated	9/167 (5.4%)	1.00 (ref)	–	1.00 (ref)	–	1.00 (ref)	–
	Not centrally heated	56/373 (15.0%)	2.79 (1.41, 5.50)	0.003	2.80 (1.42, 5.52)	0.00	1.55 (0.73, 3.28)	0.26
	Ger (Yurt)	64/398 (16.1%)	2.98 (1.52, 5.85)	0.002	3.03 (1.55, 5.96)	0.00	1.36 (0.62, 2.96)	0.48
Household income, per 100 US dollars^(c)^		–	0.94 (0.90, 0.99)	0.01	0.94 (0.90, 0.98)	0.01	0.98 (0.94, 1.02)	0.40
Home ownership	No	30/220 (13.6%)	1.00 (ref)	–	1.00 (ref)	–	–	–
	Yes	99/718 (13.8%)	1.01 (0.69, 1.48)	0.95	1.00 (0.69, 1.47)	0.98	–	
Number of people/room^(c)^		–	1.10 (1.02, 1.20)	0.02	1.11 (1.03, 1.21)	0.01	1.02 (0.93, 1.12)	0.64
Month of sampling	June–November	12/243 (4.9%)	1.00 (ref)	–	1.00 (ref)	–	1.00 (ref)	–
	December–February	54/379 (14.2%)	2.89 (1.58, 5.28)	< 0.001	2.94 (1.61, 5.38)	< 0.001	2.53 (1.23, 5.19)	0.01
	March–May	63/316 (19.9%)	4.04 (2.23, 7.31)	< 0.001	4.08 (2.26, 7.38)	< 0.001	3.31 (1.63, 6.74)	< 0.001
Number of smokers in household^(d)^	0	57/530 (10.8%)	1.00 (ref)	–	1.00 (ref)	–	1.00 (ref)	–
	1 or more	72/408 (17.6%)	1.64 (1.19, 2.27)	0.003	1.66 (1.20, 2.26)	0.002	1.29 (0.92, 1.81)	0.13
Child actively smoking	No	128/936 (13.7%)	1.00 (ref)	–	1.00 (ref)	–	1.00 (ref)	–
	Yes	1/2 (50.0%)	3.66 (0.91, 14.76)	0.07	3.84 (0.85, 17.44)	0.08	5.23 (2.70, 10.12)	< 0.001
BCG scar	Absent	27/193 (14.0%)	1.00 (ref)	–	1.00 (ref)	–	–	–
	Present	102/745 (13.7%)	0.98 (0.66, 1.45)	0.91	0.96 (0.65, 1.43)	0.85	–	
Body mass index, kg/m^2^		–	0.88 (0.82, 0.94)	< 0.001	0.88 (0.82, 0.95)	0.001	1.00 (0.89, 1.13)	0.95
Body fat, %^(c)^		–	0.95 (0.92, 0.98)	< 0.001	0.94 (0.91, 0.97)	< 0.001	0.95 (0.90, 1.00)	0.07
Household PTB contact	No	83/780 (10.6%)	1.00 (ref)	–	1.00 (ref)	–	1.00 (ref)	–
	Yes	46/158 (29.1%)	2.74 (1.99, 3.76)	< 0.001	2.72 (1.98, 3.73)	< 0.001	2.40 (1.74, 3.30)	< 0.001
De-seasonalized serum 25(OH)D^(c)^	< 10 ng/ml, *n* (%)	27/281 (9.6%)	0.63 (0.42, 0.94)	0.02	0.65 (0.43, 0.98)	0.04	0.74 (0.49, 1.10)	0.13
	≥10 ng/ml, *n* (%)	100/652 (15.3%)	1.00 (ref)	–	1.00 (ref)	–	1.00 (ref)	–

Abbreviations: 25(OH) D, 25-hydroxyvitamin D; BCG, Bacille Calmette–Guérin vaccine; CI, confidence interval; PTB, pulmonary tuberculosis; QFT, QuantiFERON®-TB Gold; ref., referent category; US, United States

^a^namely parental education, type of residence, income, number of people per room, month of sampling, presence vs. absence of indoor smokers in household, child actively smoking, body mass index, % body fat, household contact with PTB, de-seasonalized serum 25(OH) D level

^b^highest educational level attained by either parent

^c^Missing values (income, 8 missing; number of people per room, 1 missing; body fat, 5 missing; 25OHD, 5 missing)

^d^Defined as a household member other than the participating child smoking tobacco indoor

## Discussion

This large community-based cross-sectional study identifies household contact with an infectious index case and winter or spring season as risk factors for tuberculosis progression in Mongolian schoolchildren. Risk of active TB was not associated with age, sex, markers of lower socio-economic status, exposure to environmental tobacco smoke, absence of a BCG scar or vitamin D deficiency.

Our finding that ‘exogenous’ risk factors such as household exposure to an index case and season were associated with tuberculosis progression is at variance with the paradigm that risk of disease progression is primarily associated with ‘endogenous risk factors’ [4, 5]. Increased risk of disease in household contacts is likely to reflect a greater intensity of exposure and a higher inoculum, which has been reported to associate with increased risk of progression in both humans [20] and non-human primates [21]. The influence of season on tuberculosis incidence has been widely reported, with the majority of studies showing a peak in disease transmission during winter, followed by a peak of active disease in spring and summer [22]. Seasonal variation in vitamin D status has been proposed as contributing this phenomenon; however, the lack of an independent association between vitamin D deficiency and risk of active disease in the current study suggests that, in Mongolia at least, this effect is vitamin D-independent. Further research to investigate mechanisms by which seasonal variation impacts on risk if tuberculosis disease progression is warranted. We also found that active cigarette smoking was an independent risk factor for disease progression; however, the number of children actively smoking cigarettes was extremely small, and the 95% confidence intervals for the risk ratio are correspondingly very wide: this finding should therefore be interpreted with caution.

Our study has several important null findings. The lack of association between vitamin D deficiency and risk of active tuberculosis contrasts with findings from several studies conducted in adults [23–25], but is consistent with at least one other study conducted in children [26]. The lack of protection associated with presence of a BCG scar is at variance with findings of a meta-analysis reporting a 50% reduction in risk of TB disease with BCG vaccination [27]; by providing new data from Mongolia, our study adds to the literature suggesting significant global variation in BCG efficacy for the prevention of active TB [28]. Although there was a statistically significant difference in median IFN-γ concentration in supernatants of antigen-stimulated whole blood between cases and controls, there was a high degree of overlap in values; thus, our findings support the consensus that IGRA cannot be used to differentiate active tuberculosis from latent infection [29].

Our study has several strengths. Our sample size was large, giving us ample power to detect modest effects for the risk factors investigated. We used the QuantiFERON test, rather than the Tuberculin Skin Test (TST), to detect MTB infection in study participants. This minimized potential for false-positive results to arise as a consequence of sensitization to BCG or environmental mycobacteria. By restricting our analysis to sensitized individuals, we were able to specifically examine factors associated with progression from latent to active disease; a study investigating risk factors for active TB in which controls comprise a mixed population of sensitized and un-sensitized individuals would have been unable to dissect out risk factors for disease progression (reported here) from risk factors for acquisition of infection (reported previously [6]).

A significant limitation of our study was the lack of facilities for mycobacterial culture; diagnosis of active tuberculosis in children participating in our study therefore rested on clinical and radiological features alone. Tuberculosis in children is typically paucibacillary, however, and the majority of cases are not microbiologically confirmed even where culture facilities are available [30]. The proportion of infected household contacts found to have active TB in our study was 29.1%, which is intermediate between the figures of 14.7 and 38.0% reported from South African and Alaska, respectively [5, 31]; this broad similarity argues against significant under- or over-diagnosis of active disease on our part. By contrast, the overall prevalence of tuberculosis disease that we report in QFT-G-positive children (13.8%) is significantly higher than that reported elsewhere in continental East Asia (e.g. 4.6% prevalence of active tuberculosis in TST-positive Tibetan refugee schoolchildren [32]; 0% reported in Chinese schoolchildren [33]). We cannot exclude the possibility that associations observed may be due confounding: this limitation is inherent in the observational design of our study. However, the associations with household exposure, season and active smoking reported here are all clinically plausible, and they withstood adjustment for several potential confounders. A final limitation is data on HIV serostatus were not available; however, HIV infection in Mongolia has been reported to have a very low prevalence (0.02% [10]).

## Conclusions

This large cross-sectional community-based study identifies household contact with an index case of pulmonary TB and winter and spring season as being independently associated with risk of active tuberculosis in Mongolian schoolchildren. Our findings highlight the potentially high yield of household contact tracing to identify children with active tuberculosis in low- and middle-income countries [12].

## Data Availability

The datasets generated and/or analysed during the current study are not publicly available as ethical permission for this has not been granted. An anonymized dataset is available from the corresponding author on reasonable request.

## Abbreviations

aRR: Adjusted Risk Ratio
BCG: Bacillus Calmette–Guérin
BMI: Body mass index
CI: Confidence interval
CV: Coefficient of Variation
DEQAS: Vitamin D External Quality Assessment Scheme
GEE: Generalized Estimating Eqs.
HIV: Human immunodeficiency virus
ICMJE: International Committee of Medical Journal Editors
IFN-γ: Interferon-Gamma
IGRA: Interferon-Gamma Release Assays
IRB: Institutional review board
IU/ml: International Units Per Millilitre
LOQ: Limit of quantitation
MTB: *Mycobacterium tuberculosis*
NCCD: National Centre for Communicable Disease, Mongolia
PAF: Population Attributable Fraction
PTB: Pulmonary Tuberculosis
QFT-G: QuantiFERON®-TB Gold
TB: Tuberculosis
TST: Tuberculin Skin Test
VDSP: Vitamin D Standardization Program
